# Boosting LNP Performance: Higher Concentrations of Lipid Mixtures Improve In Vivo Gene Expression and Storage Stability

**DOI:** 10.3390/pharmaceutics18010050

**Published:** 2025-12-30

**Authors:** Blerina Shkodra, Ashish Muglikar, Janani Thangapandian, Matthias Schumacher, Burcu Binici, Yvonne Perrie

**Affiliations:** 1 Leon-nanodrugs GmbH, Am Klopferspitz 19, 82152 Planegg, Germany; j.thangapandian@leon-nanodrugs.com (J.T.); m.schumacher@leon-nanodrugs.com (M.S.); 2 Strathclyde Institute of Pharmacy and Biomedical Sciences, University of Strathclyde, 161 Cathedral Street, Glasgow G4 0RE, UK; muglikar11ashish@gmail.com (A.M.); burcu.eryilmaz@strath.ac.uk (B.B.); yvonne.perrie@strath.ac.uk (Y.P.)

**Keywords:** mRNA therapeutics, mRNA vaccines, mRNA-LNPs, confined jet-impingement, process optimization, process intensification, LNP stability, LNP morphology, FR-JET^®^

## Abstract

**Background**: An efficient formulation of lipid nanoparticles (LNPs) is often considered crucial in the successful development of nucleic acid therapeutics. This study explores the impact of varying the lipid and payload concentrations as starting materials on key LNP properties. **Results**: The outcomes of the study revealed that the desired particle properties could be retained even at a starting lipid mixture concentration of 70 mg/mL. Particle size remained largely unchanged despite changes in lipid mixture concentration, with polydispersity index values below 0.2. CryoTEM analysis revealed that LNPs prepared using higher lipid mixture concentrations were more uniform and more abundant in solid core morphologies. Buffer composition was shown to influence the LNP particle size, surface charge, and gene expression, as well as storage stability. In vivo studies in mice showed enhanced gene expression and biodistribution for LNPs formulated at higher lipid and RNA concentrations, with LNPs in Tris-sucrose eliciting superior gene expression compared to LNPs in PBS. **Conclusions**: This study demonstrated that intensified mixing processes based on confined jet-impingement allow the use of elevated starting material concentrations in LNP formulations, resulting in improved biological performance and stability of mRNA-LNPs, as well as enhanced scalability and throughput.

## 1. Introduction

The rapid advancement of novel messenger RNA (mRNA) vaccines for COVID-19 has drawn significant attention to lipid nanoparticles (LNPs) as a promising technology in drug formulation [1]. Market-approved LNP formulations have quickly emerged as the gold standard non-viral delivery system to encapsulate RNA material intended for applications in infectious diseases, as well as cell and gene therapies (CGTs) [2]. In contrast to other lipid-based nanoformulations—including liposomes, solid lipid nanoparticles (SLNs), nanostructured lipid carriers (NLCs) [3]—LNPs have assumed a more central role as delivery systems for biological molecules by offering adaptable and scalable manufacturing. Detailed comparisons of the structural features of LNPs versus other carriers, as well as the pharmacokinetics and the functional roles of individual lipid components, have been well described in the literature [3,4,5,6,7,8,9,10].

Besides the ability of LNPs to protect fragile biomolecules and ensure efficient cellular delivery, one of their most relevant advantages lies in enabling the rapid translation of therapies from lab to clinic, thus making them a key enabler in advancing novel therapies [11,12]. Consequently, current forecasts estimate that by 2035, 61% of the LNP market will be focused on oncological indications, largely due to their suitability for repeat dosing [13]. The recent success of the mRNA-based CRISPR therapy in treating hyperammonaemia in an infant further underlines the unique position of LNPs as effective delivery vehicles for nucleic acid-based therapies, highlighting their growing clinical potential [11]. However, despite the growing body of scientific research demonstrating the efficacy of novel therapeutics enabled by LNP technology [14], there is a clear consensus within the industry that the primary bottlenecks are no longer scientific [15]. Rather, challenges stem from unoptimized manufacturing processes, operational inefficiencies, and limited automation [16].

**Figure 1:**
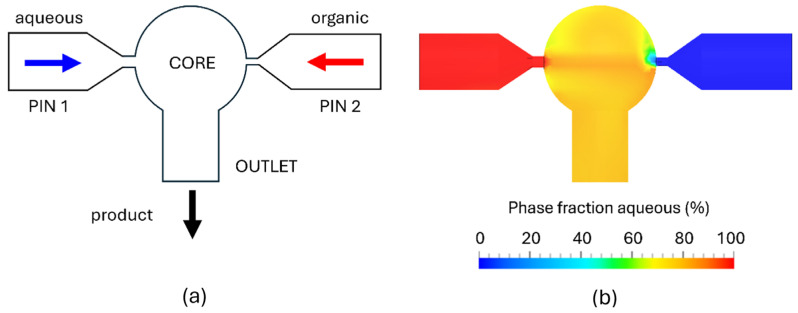
Schematic of the FR-JET^®^ modular mixer showing the internal geometry, comprising a spherical mixing chamber, 2 inlet pinholes (PIN 1 and 2) on opposing sides, and an outlet opening (**a**). CFD simulation of the phase distribution during steady-state operation (TFR = 80 mL/min) showing fully homogeneous mixing of aqueous and organic phases within the spherical chamber (**b**).

The principal technologies currently in use for mRNA-LNP manufacturing include microfluidic (MF) mixers, T-shaped mixers (often classified under confined impinging jet mixers (CIJM)), and CIJM systems with a dedicated mixing chamber [17]. MF mixers operate under laminar flow conditions, where mixing occurs predominantly through molecular diffusion. Some variations introduce secondary flow structures (e.g., herringbone grooves) to enhance mixing. MF mixers are ideal for low-volume formulations, but suffer from a high risk of clogging, poor throughput scalability, and limited adaptability to different formulation requirements [18,19]. T-mixers and traditional CIJM designs can support higher flow rates, yet their fixed geometries lead to shifts in mixing behavior when operating conditions change, resulting in inconsistent scale-up performance [19,20,21]. In contrast, the FR-JET^®^ modular mixer provides a confined mixing environment with geometry-defined flow conditions that remain stable across scales (Figure 1). Its modular architecture enables deliberate control of the mixing regime, reducing the variability typically encountered with conventional mixers during process transfer and scale-up. However, despite the variety of mixers and technologies available in the market today, challenges in LNP manufacturing persist, particularly in areas such as product throughput, scale-up, and automated workflows [22,23], which continue to demand further innovation and sustained investment to unlock the full clinical potential of LNP-based therapies. To address these bottlenecks, we explored a potential strategy for process intensification using the FR-JET^®^ modular mixer with the aim of improving manufacturing efficiency.

In this study, we investigated process intensification as a strategy to improve product throughput and facilitate scale-up, specifically by examining the effects of increasing the initial lipid mixture and RNA concentrations during LNP formulation. Increasing the concentration of the starting materials during the formulation of LNPs has relevant implications in facilitating efficient and scalable manufacturing processes [24]. In the context of commercial-scale production, formulating LNPs using higher concentrations of starting materials may offer several key advantages in terms of cost, operational efficiency, and occupational safety. By increasing the concentrations of starting materials (i.e., lipids and RNA), the process requires less organic solvent for lipid dissolution and smaller volumes of both starting and final buffers, leading to a more efficient use of materials. Moreover, smaller buffer volumes simplify handling and reduce the logistical burden of managing large containers—an issue that becomes increasingly challenging at larger manufacturing scales. In addition, working with smaller volumes of organic solvents improves occupational safety by limiting operator exposure and reducing handling-related risks. Furthermore, the use of more concentrated starting materials results in a more concentrated LNP product after mixing, potentially shortening or eliminating the initial concentration step typically required during tangential flow filtration (TFF), thus allowing a direct transition to buffer exchange. The final LNP solution can subsequently be diluted to the desired target concentration. Therefore, process intensification achieved simply by formulating at higher starting material concentrations may offer a practical way to simplify and shorten the overall manufacturing process, enhancing both operational efficiency and scalability.

Although introducing higher starting material concentrations in the manufacturing process can support process intensification, this approach is seldom reported in the literature. A likely limiting factor is the lack of efficient mixing technologies that can preserve product quality at elevated concentrations and prevent material loss. For example, the preparation of LNPs at higher lipid mixture concentrations using a microfluidics-based mixing technology has been shown to result in increased particle size and reduced encapsulation efficiency (EE) [25]. Moreover, based on published patents and patent applications describing the use of conventional T-mixing in the commercial manufacturing of LNP-based drug products—specifically Comirnaty^®^, Onpattro^®^, and Spikevax^®^—the lipid mixture concentrations used in these processes were 49.5 mg/mL, 30 mg/mL, and below 25 mg/mL, respectively [26,27,28]. Meanwhile, most peer-reviewed studies from academic research report the preparation of LNPs using even lower initial lipid mixture concentrations, typically below 15 mg/mL [29,30,31,32,33,34].

Therefore, the aim of this study was to explore the feasibility of formulating LNPs with a lipid mixture concentration of above 50 mg/mL and evaluate the effect of this process parameter on the physico-chemical properties of the resulting particles as well as their biological performance. Moderna’s Spikevax^®^ formulation was selected as the LNP model, as previous studies have demonstrated that SM-102-containing LNPs exhibited higher gene expression and better long-term stability compared to LNPs containing other ionizable lipids (e.g., ALC-0315, CKK-E12) [35,36]. The study was conducted in two parts. In the first part, feasibility experiments were performed to evaluate the formulation of LNPs across a range of lipid mixture concentrations (from 5 to 70 mg/mL), using polyadenosine (poly(A)) as a surrogate payload. In the second part, LNPs encapsulating Firefly luciferase mRNA (Fluc-mRNA) were prepared at lipid mixture concentrations of 15, 45, and 70 mg/mL, corresponding to 0.2, 0.7, and 1.0 mg/mL mRNA, respectively. LNPs were formulated at a total flow rate (TFR) of 30, 60, and 80 mL/min and dialyzed in two different buffers—phosphate-buffered saline (PBS) and Tris-sucrose. The resulting formulations were investigated for particle size distribution, EE, and particle morphology, as well as in vitro and in vivo gene expression. In addition, the effect of lipid mixture concentration and buffer composition on storage stability at 2–8 °C and 20 °C was assessed by monitoring changes in particle size and polydispersity index (PDI) over a period of six months. Our findings indicate that enabling LNP formulation at higher lipid mixture concentrations not only supports process intensification but also improves particle properties and their biological performance in vivo.

## 2. Materials and Methods

### 2.1. Materials

1,2-Distearoyl-sn-glycero-3-phosphocholine (DSPC) was purchased from Lipoid (Ludwigshafen, Germany). 1,2 dimyristoyl-rac-glycero-3-methoxypolyethylene glycol-2000 (DMG-PEG 2000) was obtained from Avanti polar lipids (Alabaster, AL, USA). Cholesterol, Poly(A), and sodium citrate tribasic dihydrate were obtained from Sigma-Aldrich (Gillingham, UK). The ionizable lipid heptadecan-9-yl 8-((2-hydroxyethyl)[6-oxo-6-(undecyloxy)hexyl]amino) octanoate (SM-102) was purchased from Broadpharm (San Diego, CA, USA). EZ Cap firefly luciferase mRNA (5-moUTP) was obtained from Apexbio (Houston, TX, USA). Ethanol and sucrose were obtained from VWR chemicals. Tris-HCl buffer (1 M), phosphate-buffered saline (100 mM; pH 7.4), MOPS buffer, Alamar blue cell viability reagent, SYBR green RNA gel stain, and Ultrapure Agarose were procured from Fisher scientific (UK). VivoGlo Luciferin in vivo grade and the One-glo luciferase assay system were purchased from Promega (Southampton, UK). The HEK 293 cell line was obtained from ATCC (Manassas, VA, USA). Lipofectamine 3000, 1,1′-dicotadecyl-3,3,3′,3′-tetramethylindocarbocyanine iodide (DiR), and trypsin EDTA (0.25%) were purchased from Thermo Fisher (Waltham, MA, USA). Amicon^®^ Ultra-15 Centrifugal filter units (100 K) and dialysis tubing cellulose membrane (14,000 K) were purchased from Merck, KGaA (Darmstadt, Germany). The other solvents and chemicals utilized were of analytical grade, and deionized water was provided by an in-house system.

### 2.2. Computational Fluid Dynamics

Computational fluid dynamics (CFD) simulations were performed using the cloud-based SimScale^®^ platform (SimScale GmbH, Munich, Germany, www.simscale.com, accessed on 29 November 2025). The FR-JET^®^ geometry and mixer configuration were implemented as defined in the experimental setup, and steady-state, as well as transient flow fields, were computed using the built-in incompressible turbulence solvers. Mesh refinement and solver settings followed SimScale’s standard best-practice guidelines to ensure numerical stability and convergence.

### 2.3. LNP Preparation

Preparation of LNPs was carried out using the FR-JET^®^ modular mixer (Leon-nanodrugs GmbH, Planegg, Germany) based on jet-impinging principles that enable a bottom-up approach for the synthesis of nanoparticles. The FR-JET^®^ modular mixer is a modular system featuring a spherical mixing chamber and pinholes, both available in various diameters to suit different processing requirements. In this experiment, a spherical chamber of 2 mm and pinhole diameters of 200 and 100 µm were used for the aqueous phase (nucleic acid) and the solvent phase (lipids), respectively. Poly(A) and Fluc-mRNA were used as payload models encapsulated in the LNPs. The size of poly(A) and Fluc-mRNA used in the formulation was 700–3500 kDa and 1921 nucleotides in length, respectively. The lipid composition consisted of SM-102 (ionizable cationic lipid), cholesterol (structural lipid), DSPC (helper lipid), and DMG-PEG2000 (PEGylated lipid) at a molar percentage ratio of 50/38.5/10/1.5, dissolved in ethanol. All LNPs, regardless of the lipid mixture concentration, were formulated at a nitrogen/phosphate (N/P)—nitrogen from the ionizable lipid and phosphate from the nucleic acid—ratio of 6 and a flow rate ratio (FRR) of aqueous to organic phases of 3:1. The lipid mixture concentration was initially varied starting from 5 mg/mL up to 70 mg/mL, with the latter identified as the limit of solubility for this lipid composition. Further, three lipid mixture concentrations of low, medium, and high—15, 45, and 70 mg/mL, corresponding to payload concentrations of 0.2, 0.7, and 1.0 mg/mL—were selected to formulate the LNPs and provide a detailed physico-chemical and biological characterization. The payload (either poly(A) or Fluc-mRNA) was initially dissolved in deionized water and then mixed with 50 mM citrate buffer (pH 4.0). The mRNA concentration was calculated based on the defined N/P ratio of 6. The N/P ratio here represents the molar ratio of ionizable nitrogen groups in the SM-102 lipid to phosphate groups in the mRNA. As SM-102 contains one ionizable amine per molecule and each nucleotide contributes one phosphate group, the mRNA molar concentration was calculated using the equation

$$\left[mRNA\right]=\frac{\left[ionizable\;lipid\right]}{6\times Nnt}$$

where [*mRNA*] is the molar concentration of mRNA; [*ionizable lipid*] is the molar concentration of the ionizable lipid SM-102; and *Nnt* is the number of nucleotides per mRNA molecule. LNPs were formulated by mixing the lipid mixture and payload solution using the FR-JET^®^ modular mixer at a TFR of 30, 60, and 80 mL/min. To remove the organic solvent from the formulations, LNPs were dialyzed in PBS (10 mM, pH 7.2) or Tris-sucrose buffer (10 mM Tris, 300 mM sucrose, pH 7.2) using dialysis cassettes (MWCO 10 kDa, cellulose membrane) while stirring at 150 rpm overnight at room temperature.

### 2.4. Particle Size Distribution and Zeta-Potential

Particle size and PDI were measured via dynamic light scattering (DLS). LNPs were characterized at a scattering angle of 173° using a Zetasizer Advance Ultra (Malvern Panalytical, Westborough, MA, USA). Particle size measurements were performed after mixing and after dialysis, diluted to 0.1 mg/mL in PBS or Tris-sucrose buffer. Electrophoretic light scattering (ELS) was used to measure the zeta-potential of the particles using the same instrument. For zeta-potential measurements, samples were diluted in water at a concentration of 0.1 mg/mL. All measurements were carried out at 25 °C in triplicate (*n* = 3). Meanwhile, the stability of the LNPs in PBS or Tris-sucrose buffer was characterized by measuring the change in particle size and PDI over a period of six months of storage at either 2–8 °C or 20 °C. LNP samples were measured via DLS at the same measurement conditions as above, at predetermined timepoints on days 0, 1, 7, 14, 21, 28, 60, 90, 120, and 180 of storage (day 0 refers to the timepoint after LNP dialysis).

### 2.5. Encapsulation Efficiency and Payload Recovery

Encapsulation efficiency was characterized with the RiboGreen assay. Measurements were carried out using the Quant-iT^TM^ RiboGreen^TM^ RNA Assay kit (Invitrogen^TM^, Thermo Fisher Scientific, Waltham, MA, USA). The Ribogreen reagent is an ultra-sensitive dye that fluoresces when bound to nucleic acids. TE buffer (10 mM pH 7.5) was used to dilute the Ribogreen reagent, LNP samples, and RNA standard solutions. For the standard curve, dilutions of RNA stock solution at 4 µg/mL and 0.4 µg/mL were prepared from the 100 µg/mL RNA standard solution provided with the reagent kit. Each standard curve was plated over two rows to give duplicate measurements. Similarly, LNP samples were diluted at a concentration of 3 µg/mL and plated into the wells. Two wells were filled with TE buffer and two wells with 2% (*w*/*v*) Triton-X, and the samples were incubated for 15 min. After incubation, 100 µL of Ribogreen reagent was added to each well and gently pipetted up and down for proper mixing. Poly(A) was quantified by measuring fluorescence (λem = 525 nm, λex = 485 nm) at a gain value of 1400 using a fluorimeter (Polarstar Omega, BMG Labtech, Ortenberg, Germany).

### 2.6. Gel Electrophoresis

Gel electrophoresis was performed to assess the integrity of the encapsulated Fluc-mRNA at a total flow rate of 80 mL/min. 200 µL of Fluc-mRNA LNP solution, corresponding to 10 µg () Fluc-mRNA, was treated with 750 µL of ethanol and 25 µL of 3 M sodium acetate(pH 5.2). Next, samples were subjected to centrifugation at 14,000 rpm for 20 min. The ethanol precipitation and centrifugation steps were repeated twice, and the Fluc-mRNA pellet was collected. The pellet was then washed with 35 µL of water treated with diethyl pyrocarbonate (DEPC) and mixed with formaldehyde dye (1:3 *v*/*v*). Subsequently, samples were denatured at 70 °C for 10 min. Sample concentrations of 0.2 µg (5–10 µL) were loaded onto 1% denatured agarose gel in 3-(morpholino) propane sulfonic acid running buffer. The gel was pre-stained with SYBR green stain and run at 90 V. The Ambion Millennium marker was used as the molecular weight standard [37]. The gel images were captured in a Gel Doc EZ imager (Bio-Rad, Hercules, CA, USA).

### 2.7. Alamar Blue Assay

The cells were seeded in a 96-well plate at a density of 10,000 cells per well and allowed to grow until confluent. Further, the medium was aspirated, and cells were transfected with 100 µL of LNPs in minimal essential medium (MEM) at lipid concentrations of 44 µg/mL, 22 µg/mL, 11 µg/mL, and 5.5 µg/mL, and incubated for 24 h. After incubation, LNPs were aspirated, and 100 µL of fresh medium was added containing 10% Alamar blue reagent. As a control, cells were treated with 1% triton in fresh medium along with Alamar blue. The fluorescence was measured after 6 h of incubation period at a wavelength of λem = 520 nm, λex = 630 nm using a UV-VIS plate reader (Promega, Madison, WI, USA, GLoMax^®^ Discover Microplate reader). The cell viability assay was repeated three times per LNP formulation. Relative cell viability% = (A test)/(A control) × 100.

### 2.8. In Vitro Transfection Efficiency and Gene Expression

For transfection efficiency studies, HEK293 (human embryonic kidney) cells were used, and luciferase activity was determined using the ONE-GLO luciferase assay system. The cells were maintained in a suitable medium and incubated as per standard conditions until they reached confluency. In a clear-bottom 96-well plate, 1 × 10^4^ cells were seeded per well and allowed to adhere for 48 h at 37 °C and 5% CO_2_. After 48 h, the media was aspirated, and cells were transfected with 100 µL of LNPs in MEM at concentrations of 2 µg/mL, 1 µg/mL, 0.5 µg/mL, and 0.25 µg/mL of Fluc-mRNA, followed by incubation for 24 h. As a control, cells were treated with Fluc-mRNA transfected with Lipofectamine 3000 following the manufacturer’s instructions. The next day, 100 µL of ONE-Glo luciferase reagent was added to the wells, and luminescence was measured using a UV-VIS plate reader (Promega, Madison, WI, USA, GLoMax^®^ Discover Microplate reader). Statistical analysis was performed using one-way ANOVA to evaluate the differences between groups. Post hoc comparisons were conducted using the paired *t*-tests to determine pairwise group differences.

### 2.9. In Vivo Biodistribution and Gene Expression

The expression and biodistribution profiles of the encapsulated Fluc-mRNA in mice were investigated using an in vivo imaging system (IVIS) (Revvity, Waltham, MA, USA). Male BALB/c mice (8–10 weeks old) were obtained from the Biological Procedures Unit at the University of Strathclyde, Glasgow, with five mice per group. All protocols were subjected to ethical review and conducted in a designated establishment.

Mice were injected via an intramuscular (i.m.) route in both legs with 5 µg of Fluc-mRNA LNPs labeled with a fluorescent dye 1,1′-dioctadecyl-3,3,3′,3′-tetramethylindotricarbocyanin iodide (DiR) (excitation/emission: 754/778 nm). Mice were anesthetized in a ventilated anesthesia chamber with 3% isoflurane in oxygen and then imaged using a DiR filter to assess LNP biodistribution. Subsequently, the mice received D-luciferin (150 mg per kg) subcutaneously, and 10 min later, bioluminescence imaging was performed using an open filter with an auto-exposure setting (almost 15 min elapsed between LNP i.m. injection and bioluminescence imaging). These imaging sessions were repeated 6, 24, and 48 h after i.m. injection of LNPs. Image capture and data analysis were performed using Living Image software^®^ 4.7.3. To account for tissue autofluorescence and background noise, data normalization was performed. The raw signal intensity values (total flux for bioluminescence and radiant efficiency for fluorescence) of each experimental region of interest were divided by the corresponding signal obtained from untreated control animals. Consequently, quantitative results are expressed as fold change relative to the control (arbitrary units, a.u.) and are presented as mean ± standard error of the mean (SEM).

### 2.10. Imaging Using Cryogenic Transmission Electron Microscopy (cryoTEM)

All samples were prepared following standardized procedures to ensure consistency and optimal imaging conditions. Frozen samples were first thawed at room temperature, while refrigerated samples were maintained on ice throughout the preparation process. A volume of 4 μL of undiluted sample was applied to an ATEM regularly spaced holey carbon-copper grid (ATEM LNPCFoil Grids, Remscheid, Germany) under a water-saturated atmosphere at 4 °C and incubated for one minute using a Thermo Fisher Vitrobot Mark IV system (Thermo Fisher Scientific, Waltham, MA, USA). Excess liquid was removed by blotting, leaving a thin film—several hundred nanometers thick—on the grid surface. The blotted grids were immediately vitrified by plunge-freezing in liquid ethane at −180 °C. Vitrified samples were subsequently stored in sample-specific containers submerged in liquid nitrogen (LN_2_) until further use in cryo-electron microscopy. Prepared grids were transferred into a 200 kV Thermo Fisher Glacios cryoTEM (Thermo Fisher Scientific, Waltham, MA, USA) equipped with a Falcon IVi direct electron detector and operated via Thermo Fisher EPU software 3.12. An overview image (Atlas) of each grid was acquired at low magnification (150×) to facilitate assessment. Trained operators evaluated each Atlas to identify areas exhibiting ideal ice thickness, targeting regions with an estimated thickness of 200–300 nm. Suitable imaging regions and appropriate defocus settings were then manually defined within these selected areas. High-resolution image acquisition was performed at approximately 45,000× magnification under low-dose conditions using dose fractionation on the Falcon IVi detector. The resulting dose-fractionated raw micrographs were processed through frame alignment to correct for beam-induced motion and enhance structural detail. The aligned and computationally summed images were retained for further data analysis. All preprocessed micrographs were analyzed using a version-controlled, proprietary machine learning (ML) model developed by ATEM for automated evaluation and interpretation of imaging data.

## 3. Results

SM-102-containing LNPs encapsulating poly(A) or Fluc-mRNA were prepared using the FR-JET^®^ modular mixer with a chamber size of 2 mm and a pinhole combination of 200 µm for the payload solution and 100 µm for the lipid solution. To investigate the feasibility of particle preparation at higher lipid mixture and mRNA concentrations, LNPs encapsulating poly(A) were first prepared across a broad range of lipid mixture concentrations from 5 to 70 mg/mL at a TFR of 15 mL/min. Next, formulation parameters with lipid mixture concentrations of 15, 45, and 70 mg/mL (corresponding to 0.2, 0.7, and 1.0 mg/mL payload concentrations) were selected and prepared at three TFRs—30, 60, and 80 mL/min. Lastly, Fluc-mRNA LNPs prepared using the selected formulation and process parameters were investigated for the integrity of the encapsulated Fluc-mRNA, as well as for cell viability and gene expression in vitro and in vivo.

### 3.1. Effect of Lipid Mixture Concentration on LNPs

A preliminary set of LNP formulations encapsulating poly(A) were produced across a broad range of lipid mixture concentrations to identify the failure threshold for this process parameter. A lipid mixture concentration of 5 mg/mL was set as the lowest concentration, whereas 70 mg/mL was set as the highest concentration due to the solubility limit of this lipid mixture in ethanol. It should be noted that lipid mixtures containing alternative combinations of lipids display different solubility limits. In general, we observed that particle size decreased with increasing lipid mixture concentration for both LNPs dialyzed in PBS and Tris-sucrose (Table 1). The particle size of LNPs in PBS decreased from 86 nm at a lipid mixture concentration of 5 mg/mL to 76 nm at 70 mg/mL (Table 1). Meanwhile, the particle size of LNPs in Tris-sucrose was larger compared to LNPs in PBS, starting at 108 nm for LNPs with a lipid mixture concentration of 15 mg/mL and 103 nm for 70 mg/mL (Table 1). The difference in particle size was likely a result of the ionic strength of the buffers, i.e., the additional 10% (*w*/*v*) sucrose in Tris-sucrose LNPs [36,38,39,40]. Moreover, the effect of lipid mixture concentration was also evident on the surface charge of the LNPs, going from negative to positive zeta-potential values with increasing lipid mixture concentration (Table 1). The buffer composition also affected the surface charge of the particles [32,39]. LNPs dialyzed in Tris-sucrose displayed positive zeta-potential values of +25 to +35 mV (Table 1), whereas LNPs dialyzed in PBS showed near-neutral zeta-potential values for LNPs with lipid mixture concentrations up to 30 mg/mL and around +10 mV for LNPs with lipid mixture concentrations of 40 to 70 mg/mL (Table 1). The positive surface charge of the LNPs in Tris-sucrose originates from the protonated cationic nitrogen center [32], whereas the negative or near-neutral charge of LNPs in PBS originates from the phosphate anions, which reduce the surface charge of the LNPs. The EE was, on average, 96.4% and 96.8% for LNPs in PBS and Tris-sucrose, respectively, indicating a robust mixing process (Table 1).

### 3.2. Effect of Lipid Mixture Concentration and TFR on LNPs

In the next set of formulations, LNPs were prepared at selected low, medium, and high lipid mixture concentrations of 15, 45, and 70 mg/mL and poly(A) concentrations of 0.2, 0.7, and 1.0 mg/mL, respectively. The results indicated that LNPs with particle sizes of ± 10 nm were formulated regardless of the tested TFR of 30, 60, or 80 mL/min (Table 2). In general, LNPs dialyzed in Tris-sucrose were larger in size compared to LNPs dialyzed in PBS due to the higher ionic strength of Tris-sucrose buffer [39]. LNPs dialyzed in Tris-sucrose exhibited positive zeta-potential values (+10 to +30 mV), whereas LNPs dialyzed in PBS exhibited near-neutral values for LNPs with a lipid mixture concentration of 15 mg/mL and slightly positive values of around +5 to +9 mV for LNPs with lipid mixture concentrations of 45 and 70 mg/mL (Table 2).

EE for LNPs prepared at a TFR of 30 mL/min was between 92% and 94% (Table 2). Furthermore, EE for LNPs with a lipid mixture concentration of 15 mg/mL prepared at TFR of 60 and 80 mL/min was slightly lower (90 to 93%) compared to the EE of LNPs with a lipid mixture concentration of 45 and 70 mg/mL (92 to 95%). Moreover, our results revealed that increasing the lipid mixture concentration improved the recovery of the payload in the LNPs. This aligns with findings reported by Schober et al., who, despite working with lower lipid mixture concentrations of around 7 to 8 mg/mL—which are significantly lower than those enabled here by the FR-JET^®^ modular mixer—demonstrated that increasing lipid mixture concentration within their formulations led to a marked improvement in mRNA loading efficiency of up to 82%, indicating a considerable reduction in mRNA loss during the process [2].

### 3.3. Effect of Lipid Mixture Concentration on Particle Morphology

**Figure 2:**
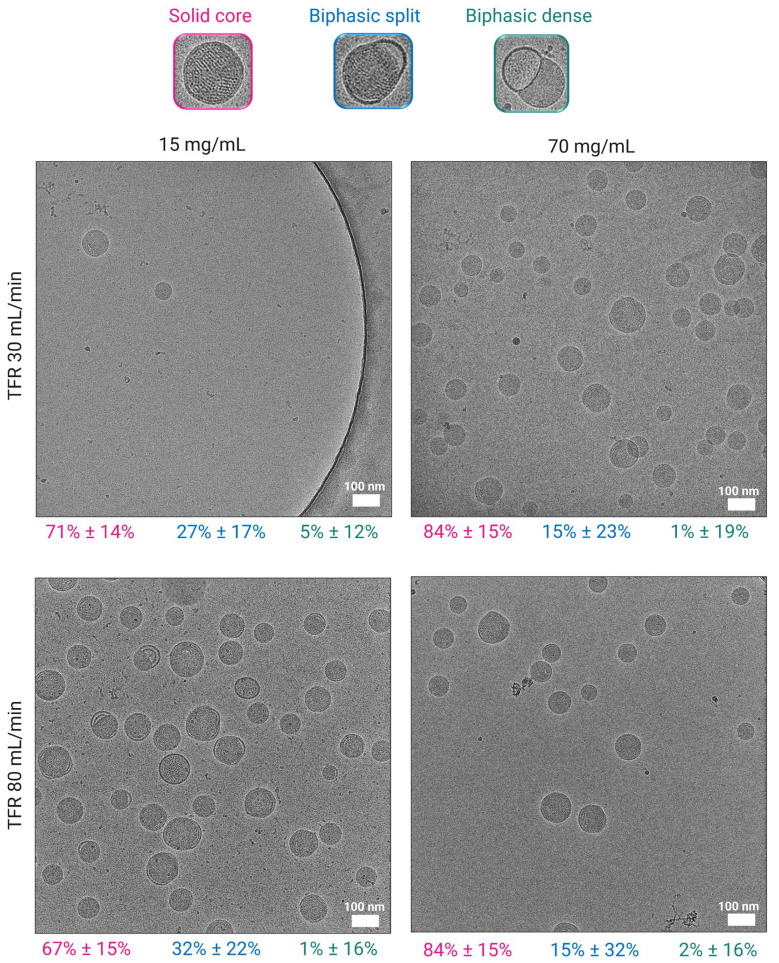
Morphological analysis of LNPs dialyzed in PBS using cryoTEM. Images of LNPs formulated at 15 mg/mL and 70 mg/mL at TFRs of 30 and 80 mL/min, scale bars 100 nm. Morphology distribution of LNPs as a percentage is shown below the images (pink—solid core; blue—biphasic split; green—biphasic dense). Biphasic split refers to a smaller flat-like bleb splitting from the particle surface, whereas biphasic dense refers to larger blebs protruding from the core of the particle.

CryoTEM images illustrated the morphological characteristics of LNPs prepared at lipid mixture concentrations of 15 mg/mL and 70 mg/mL, under TFRs of 30 mL/min and 80 mL/min, and dialyzed in PBS (Figure 2). The corresponding quantitative analysis revealed that across all conditions, LNPs predominantly exhibited solid core structures, accounting for 67 to 84% of the observed morphologies (Figure 2). Notably, increasing the lipid mixture concentration to 70 mg/mL led to a significant rise in the proportion of solid cores, regardless of the TFR. Chander et al. previously reported that increasing the molar ratio of helper lipids in the lipid mixture composition promoted the formation of solid core morphologies [41]. However, our findings suggest that a similar outcome can be achieved without altering the molar ratios, but simply by increasing the lipid mixture concentration, at least when an intensified mixing process is used. In contrast, LNPs formulated at 15 mg/mL showed a lower percentage of solid cores (~70%) and a higher distribution of biphasic-split structures (also known as blebs) compared to LNPs formulated at 70 mg/mL. The larger bleb formations (depicted here as biphasic dense) remained consistently low (1 to 5%) across all conditions (Figure 2). These results indicate that higher lipid mixture concentration promotes the formation of more uniform and structurally compact LNPs, while the impact of TFR appeared to be less pronounced in influencing morphological outcomes. Furthermore, the observed results of morphological differences between LNPs with low vs. high lipid mixture concentrations may explain our findings, where superior in vivo gene expression was observed with LNPs formulated at higher lipid mixture concentrations reported in Section 3.8. The presence of spherical solid-core morphologies appears to enhance this effect, as demonstrated by Chen et al., who also reported that spherical LNPs exhibit superior transfection efficiency [42]. These findings further emphasize the critical influence of LNP physico-chemical properties on the therapeutic efficacy of the encapsulated agent.

### 3.4. Effect of Lipid Mixture Concentration and TFR on Payload Integrity

**Figure 3:**
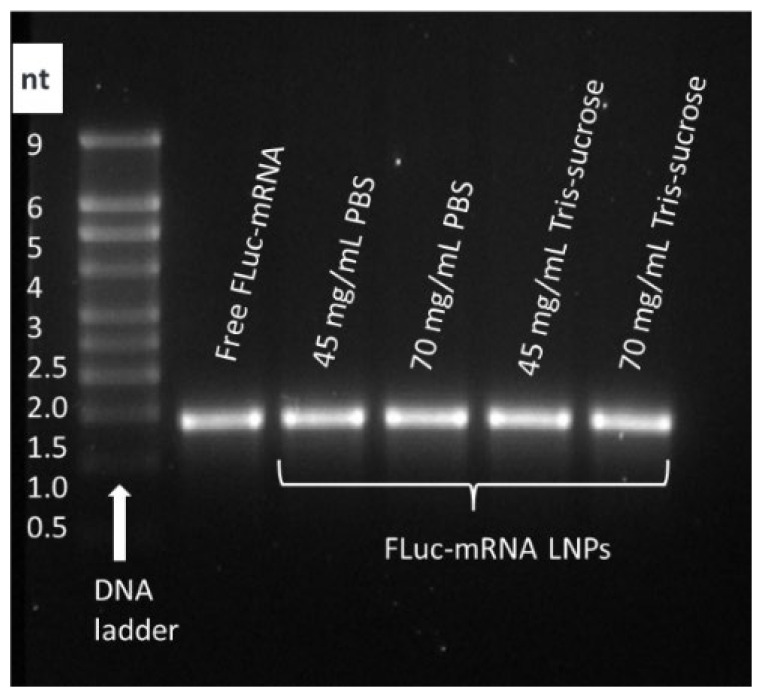
Gel electrophoresis was performed to assess the integrity of encapsulated Fluc-mRNA 1921 nucleotides in length. Fluc-mRNA LNPs were prepared at a TFR of 80 mL/min using lipid mixture concentrations of 45 and 70 mg/mL, corresponding to 0.7 and 1.0 mg/mL of Fluc-mRNA.

Gel electrophoresis was performed to investigate whether the Fluc-mRNA remains intact when processed at high starting material concentrations and at a high TFR of 80 mL/min. The Fluc-mRNA used in the formulation was 1921 nucleotides in length. The gel electrophoresis image (Figure 3) showed that the Fluc-mRNA encapsulated in the LNPs displayed distinct bands corresponding to a biomolecule of approximately 2 kb, which was comparable to the free Fluc-mRNA used as a control in this experiment. These results demonstrated that the preparation process preserved the integrity of the encapsulated Fluc-mRNA even at considerably high concentrations of materials prepared at high flow rates.

### 3.5. Cell Viability

**Figure 4:**
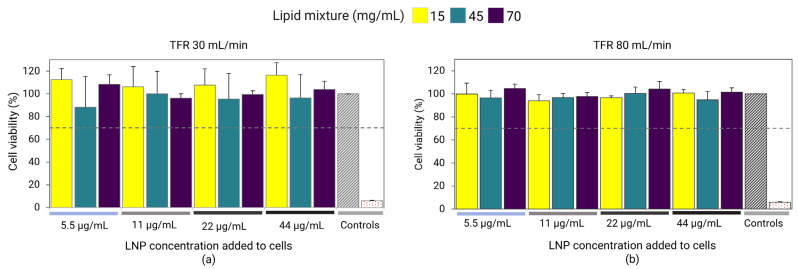
Fluc-mRNA-loaded LNPs prepared using a lipid mixture concentration of 15, 45, and 70 mg/mL at TFRs of 30 mL/min (**a**) and 80 mL/min (**b**). The LNPs were dialyzed in PBS and sterile-filtered using 0.22 µm PES. LNPs were incubated with HEK293 kidney cells at LNP concentrations of 5.5, 11, 22, and 44 µg/mL. Cell viability was determined by the Alamar Blue assay. Cell media was used as a positive control (gray diamond grid bar), whereas 1% Triton was used as a negative control (red-dotted bar). Error bars represent the standard deviation of the cell assay repeated three times per LNP formulation (*n* = 3).

Fluc-mRNA LNPs prepared at different lipid mixture concentrations were investigated for their impact on cellular viability. Results revealed that LNPs prepared at a TFR of 30 and 80 mL/min exhibited no toxic effects and cell viability was above 70% for all LNP samples, regardless of the lipid mixture concentration (Figure 4). Results showed that despite the high lipid and RNA concentrations used in the preparation, the LNPs were well tolerated and suitable for in vivo investigation.

### 3.6. In Vitro Gene Expression

**Figure 5:**
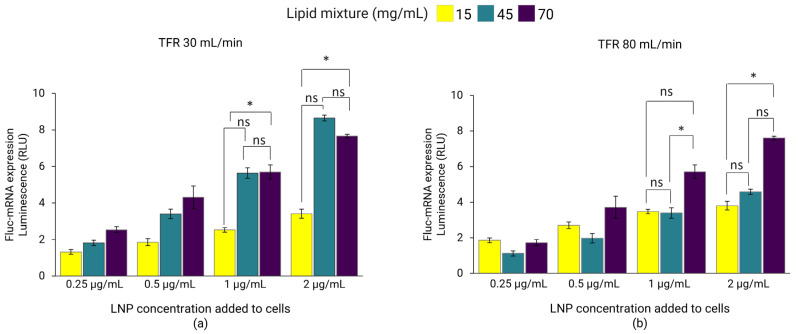
Fluc-mRNA-loaded LNPs prepared using lipid mixture concentrations of 15, 45, and 70 mg/mL at TFRs of 30 mL/min (**a**) and 80 mL/min (**b**). LNPs were dialyzed in PBS and sterile-filtered (0.22 µm PES) before incubation with HEK293 kidney cells to a Fluc-mRNA concentration of 0.25, 0.5, 1, and 2 µg/mL. The luminescence of the luciferase activity was determined using the ONE-Glo luciferase assay on a plate-reader. Error bars represent the standard deviation of the transfection assay repeated three times per LNP formulation (*n* = 3). Statistical analysis was performed using one-way ANOVA followed by paired *t*-tests to compare group differences, conducted using Excel. ns indicates not significant (*p* > 0.05). Statistical significance: * indicates *p* < 0.05.

In the first set of experiments, Fluc-mRNA LNPs dialyzed in PBS prepared at TFRs of 30 mL/min and 80 mL/min were investigated for the expression of luciferase in the HEK293 cell line. For LNPs prepared at a TFR of 30 mL/min, luciferase expression significantly improved with lipid mixture concentrations of 45 and 70 mg/mL compared to 15 mg/mL (Figure 5a). This trend correlated with the zeta-potential of the LNPs (Table 2), suggesting that LNPs with more positively charged particles might interact more strongly with cells, subsequently improving cell-uptake and gene expression [43]. This was especially evident at higher incubation concentrations of 1 and 2 µg/mL, where comparable gene expression was observed for LNPs with lipid mixture concentrations of 45 and 70 mg/mL exhibiting comparable zeta-potentials (Table 2), whereas, at lower LNP incubation concentrations (0.25 and 0.5 µg/mL), Fluc-mRNA expression did not differ significantly between formulations (*p* > 0.05). Meanwhile, for LNPs prepared at a TFR of 80 mL/min, gene expression was highest for LNPs with a lipid mixture concentration of 70 mg/mL, whereas expression was comparable for LNPs with concentration of 15 and 45 mg/mL (Figure 5b). The significant improvement in the gene expression observed with LNPs formulated at higher lipid mixture concentrations may also be influenced by their improved payload recovery (Table 2).

**Figure 6:**
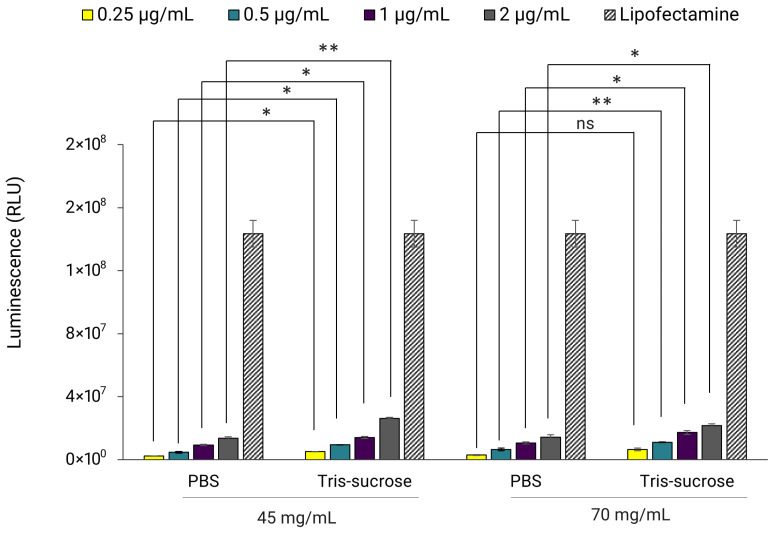
Fluc-mRNA-loaded LNPs prepared at a TFR of 80 mL/min using lipid mixture concentrations of 45 and 70 mg/mL. LNPs were incubated with HEK293 kidney cells at a Fluc-mRNA concentration of 0.25, 0.5, 1, and 2 µg/mL. The luminescence of the luciferase activity was determined with the ONE-Glo luciferase assay using a UV-VIS plate-reader. Lipofectamine™MessengerMAX™ reagent was used as a transfection control. Error bars represent the standard deviation of the transfection assay repeated three times per LNP formulation (*n* = 3). Statistical analysis was performed using paired *t*-tests to compare differences, conducted using Excel. ns indicates not significant (*p* > 0.05). Statistical significance is indicated as follows: * *p* < 0.05, ** *p* < 0.01.

In the next set of experiments, the influence of buffer on luciferase expression was examined. Here, Fluc-mRNA LNPs with lipid mixture concentrations of 45 and 70 mg/mL, dialyzed in PBS and Tris-sucrose, were incubated with HEK293 cells. The results revealed that buffer composition influenced luciferase expression, with LNPs in Tris-sucrose displaying significantly higher luminescence intensity compared to LNPs in PBS, regardless of the lipid mixture concentration (Figure 6). Again, the gene expression here correlated with the zeta-potentials of the LNPs (Table 2). LNPs in Tris-sucrose elicited more positively charged surfaces compared to LNPs in PBS, which may have improved cell internalization, and thereby gene expression. In addition, the 10% sucrose in the Tris buffer may also have contributed to the improved gene expression, aligning with other studies that demonstrated similar results when using 2–10% sucrose in their LNP formulations [36].

### 3.7. In Vivo Biodistribution

**Figure 7:**
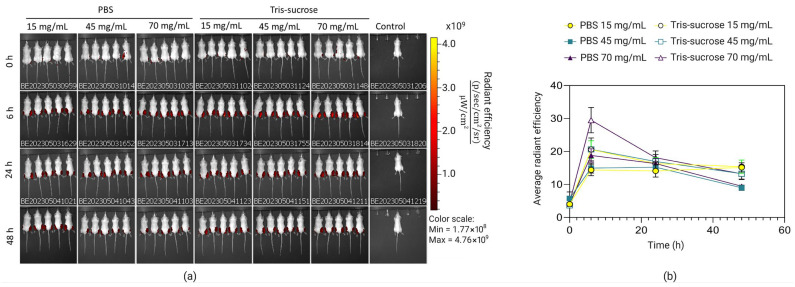
Representative IVIS images of groups of five BALB/c mice injected with DiR-labelled LNPs intramuscularly at specific timepoints following DIR-labelled mRNA, where 0 h refers to the time 15 min after injection of the LNPs (**a**). Measurements of the fluorescence of DIR in mRNA-LNPs prepared with lipid mixture concentrations of 15 mg/mL, 45 mg/mL, and 70 mg/mL and dialyzed against Tris-sucrose or PBS (**b**). Data are expressed as standard error of the mean (*n* = 3).

The effects of the lipid mixture concentration and the choice of buffer on the biodistribution and expression profile of Fluc-mRNA LNPs in BALB/c mice were tested using IVIS. Local retention was monitored longitudinally at 0 h, 6 h, 24 h, and 48 h after i.m. injection (where 0 h refers to 15 min after administration). Immediately at 0 h after i.m. injection, all LNPs displayed similar fluorescence intensities, indicating comparable initial loading at the injection site (Figure 7). Interestingly, the intensity at the injection site increased for all LNPs at 6 h. Di et al. reported a similar phenomenon for DiR-labelled LNPs [44]. The increasing fluorescence intensity over time was likely due to DiR leakage from the LNP membrane. At the 6 h peak, for the majority of formulations, neither the lipid mixture concentration nor the dialysis buffer significantly affected local retention; no significant differences were observed among the PBS groups (15, 45, 70 mg/mL) or the lower-concentration Tris-sucrose groups (15, 45 mg/mL) (*p* > 0.05). The only exception was the Tris-sucrose 70 mg/mL formulation, which exhibited significantly higher fluorescence intensity compared to its PBS counterpart (*p* < 0.05) and to the lower-concentration Tris-sucrose formulations (*p* < 0.05). This could be a result of increased interactions between cationic particles (zeta-potential, Table 2) and negatively charged physiological membranes, whereas this interaction might have been lower with neutral particles (LNPs in PBS), which elicited a lower fluorescence intensity [45]. However, this enhanced retention observed with Tris-sucrose 70 mg/mL was transient. During the clearance phase (24 h and 48 h), fluorescence intensities of all experimental groups converged, and no statistically significant differences were observed between any formulations irrespective of the buffer or lipid mixture concentration (*p* > 0.05). This indicates that while the Tris-sucrose buffer at high lipid loads maximizes initial tissue retention, the subsequent clearance rate is comparable across all groups. Additionally, some LNPs accumulated in the liver 6 h after i.m. injection and remained stable over the monitored timepoints (Supplementary Materials, Figure S1, left).

### 3.8. In Vivo Gene Expression

**Figure 8:**
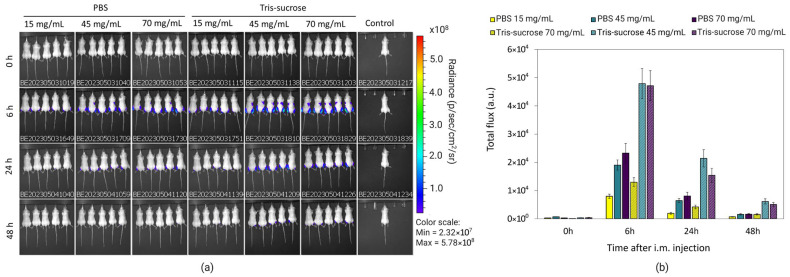
Representative IVIS images of groups of 5 BALB/c after intramuscular administration of DiR-labelled Fluc-mRNA LNPs acquired at the indicated timepoints. Time 0 h corresponds to 15 min after injection (**a**). Quantification of luciferase expression at the injection site of LNPs encapsulating Fluc-mRNA 0 h, 6 h, 24 h, and 48 h of post-injection (**b**). LNPs were prepared with lipid mixture concentrations of 15, 45, and 70 mg/mL and were purified against PBS and Tris-sucrose. Data are expressed as standard error of the mean (*n* = 3).

**Figure 9:**
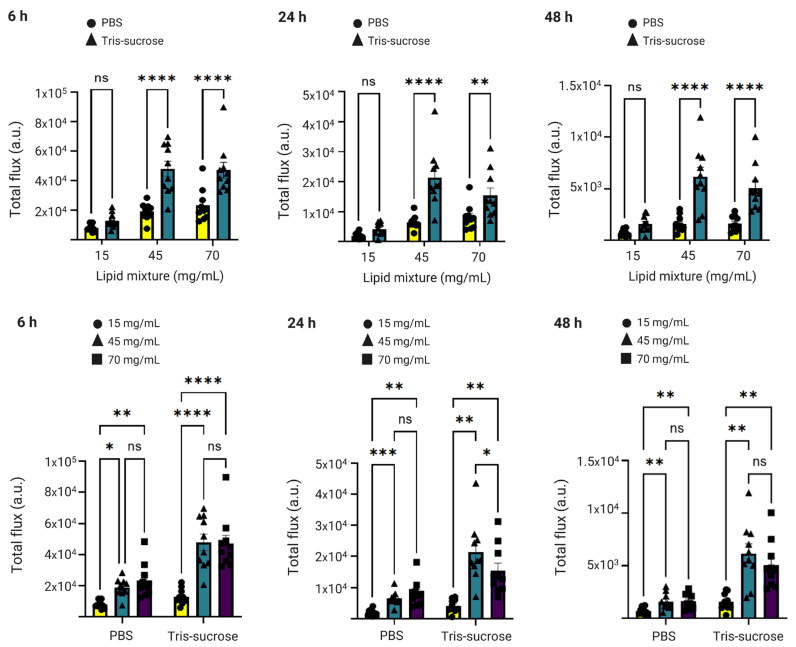
Luciferase expression at the injection site at 6 h, 24 h, and 48 h after injection of the Fluc-mRNA LNPs as a function of the lipid mixture concentration (upper panel) and buffer (lower panel). Data are expressed as standard error of the mean (*n* = 3) followed by ANOVA analysis using GraphPad Prism 10. ns indicates not significant (*p* > 0.05). Statistical significance is indicated as follows: * *p* < 0.05, ** *p* < 0.01, *** *p* < 0.001, and **** *p* < 0.0001.

The same mice were analyzed simultaneously to investigate the effects of the lipid mixture concentration and the choice of the buffer on mRNA-LNP expression efficiency. Local protein expression kinetics were evaluated by quantifying total flux (bioluminescence) over 48 h following bilateral intramuscular injections. Bioluminescence intensity increased sharply, reaching a peak at 6 h post-administration for all formulations (Figure 8b). At this 6 h peak, LNPs formulated with a lipid mixture concentration of 15 mg/mL exhibited significantly lower expression compared to LNPs with higher lipid mixture concentrations (45 and 70 mg/mL). Specifically, in the Tris-sucrose buffer group, increasing the concentration from 15 mg/mL to 45 mg/mL resulted in a highly significant increase in protein expression (*p* < 0.0001). Similarly, within the PBS group, the 15 mg/mL formulation showed significantly lower expression compared to the 45 mg/mL (*p* < 0.05) and 70 mg/mL (*p* < 0.01) formulations (Figure 9). This followed a similar trend to the in vitro expression of mRNA-LNPs in HEK293 cells reported earlier in Section 3.6. Again, the improved gene expression with LNPs of higher lipid mixture concentrations is likely related to the more positively charged LNP surfaces [2]. The impact of the dialysis buffer was highly dependent on the lipid mixture concentration. For the lowest concentration (15 mg/mL), the choice of buffer did not significantly alter the expression efficiency (*p* > 0.05). However, for the higher lipid concentration groups, the buffer played a critical role. Dialyzing LNPs against Tris-sucrose drastically increased the mRNA expression compared to PBS-dialyzed counterparts. At the 6 h peak, both the 45 mg/mL and 70 mg/mL formulations in Tris-sucrose showed extremely significant increases in total flux compared to their PBS equivalents (*p* < 0.0001) (Figure 9). Henderson et al. showed that dialyzing MC3-containing LNPs in Tris buffer instead of PBS significantly increased the in vivo mRNA expression in BALB/c mice receiving LNPs intravenously despite the LNPs having similar morphological properties [46]. Meulewaeter et al. showed cryoTEM images of C12-200 LNPs dialyzed in PBS or Tris buffer for 5 h at 4 °C [47]. Their study revealed that dialyzing LNPs in Tris buffer resulted in LNPs with a solid amorphous core, while dialyzing LNPs against PBS led to a heterogeneous morphology, including LNPs containing an aqueous compartment (blebs), which appeared to entrap mRNA. Some of these blebs transformed into liposomal structures, dissociating from the LNPs. This structural difference can be an obstacle to releasing mRNA into the cytosol, which is the target site for translating mRNA into the corresponding protein [39]. In another study, Brader et al. employed an RNA-binding dye (thionine) to stain LNPs, revealing the bleb structures, including mRNA, and observed a significant alteration in LNP morphology after overnight dialysis in a pH 5 buffer to simulate the endosome environment [48]. These bleb structures were reassociated with the lipidic body of LNPs, leaving the bleb compartment empty, whereas the mRNA remained encapsulated within a spherical particle, which could potentially delay the release of mRNA into the cytosol. The superior efficacy of the Tris-sucrose formulations was sustained over time. Even at 24 h and 48 h post-injection, the 45 mg/mL Tris-sucrose group maintained significantly higher expression levels compared to the PBS group (*p* < 0.0001). Moreover, luciferase expression quantified in the liver followed a similar trend to that observed at the injection site (Supplementary Material, Figure S1, right). In conclusion, while the mRNA expression of LNPs prepared with a low lipid mixture concentration (15 mg/mL) was not affected by the choice of buffer (*p* > 0.05), formulations with higher lipid mixture concentrations showed markedly superior expression when dialyzed against Tris-sucrose (*p* < 0.0001) [36].

### 3.9. Effect of Lipid Mixture Concentration on LNP Stability

The stability of LNP-based therapies is an imperative aspect to consider, since it dictates the storage conditions and the shelf-life of the product as well as its entire life cycle [49]. Furthermore, LNP stability is one of the main factors driving the cost and causing bottlenecks in the supply chain process of the mRNA-LNP products [35,50]. The physical stability of LNP formulations is typically assessed by monitoring the changes in the physico-chemical critical quality attributes (CQAs) (i.e., particle size and PDI), whereas the functional stability of LNPs is assessed by investigating changes in mRNA efficacy (i.e., mRNA content, mRNA integrity, and gene expression) [51]. However, according to the regulatory guidelines, stability is mainly assessed by monitoring the physical CQAs (particle size, PDI, pH, osmolality) [51,52,53,54], since changes in physical attributes typically result in aggregate formation, thereby affecting the efficacy of the mRNA-LNP products [36]. Besides long-term storage stability, controlling the particle size of LNPs between different unit operations during the manufacturing process is critical to ensure consistent product quality [34], since variations in LNP size can significantly impact the in vivo efficiency of the encapsulated payload [55].

**Figure 10:**
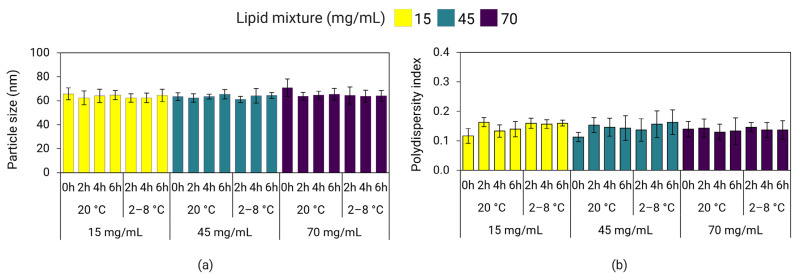
Hold-time stability of LNPs containing 25% ethanol prepared at a TFR of 30 mL/min and stored at 2–8 °C and 20 °C. Particle size (**a**) and PDI (**b**) of poly(A)-LNPs were measured on DLS after a hold-time of 2, 4, and 6 h. Error bars represent the standard deviation between formulation replicates (*n* = 3).

Here, we investigated the effect of the lipid mixture concentration on short- and long-term storage stability of poly(A)-LNPs stored at 2–8 °C and 20 °C. Short-term stability (also known as hold-time stability) of the LNPs immediately after mixing was investigated by measuring variations in particle size and PDI after 2, 4, and 6 h of hold-time. Long-term stability was investigated by measuring variations in particle size and PDI on days 0, 1, 7, 14, 21, 28, 60, 90, 120, 150, and 180 of storage. LNPs with a particle size of ≤120 nm and PDI ≤ 0.25 were considered stable. Figure 10 shows that LNPs after mixing, prepared without in-line dilution and containing 25% ethanol, maintained their particle size and PDI for at least up to 6 h of hold-time when stored at 2–8 °C and 20 °C. Although longer timepoints were not evaluated in this experiment, a 6 h hold-time stability would be considered sufficient to accommodate transfer of the intermediate LNP product from the mixing step to the next unit operation of buffer exchange, as typically required during large-scale manufacturing processes. In contrast, another study reported that LNPs prepared via nanoprecipitation increased in size by about 25 nm after 5 h of hold-time [56].

**Figure 11:**
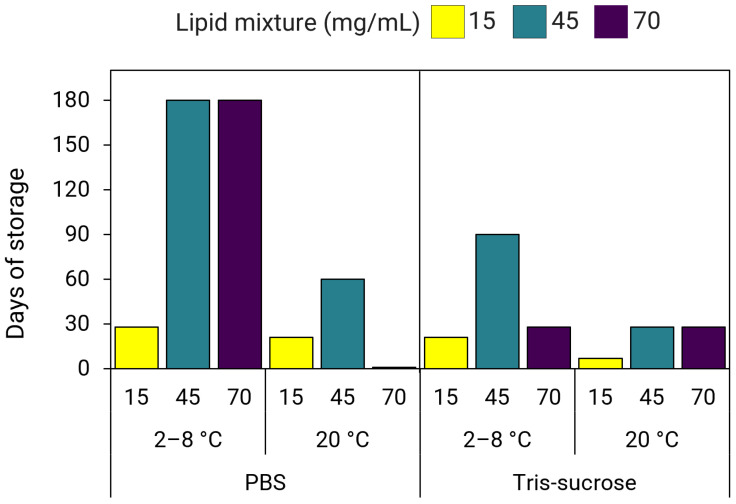
Long-term storage stability of LNPs prepared using lipid mixture concentrations of 15, 45, and 70 mg/mL. LNPs dialyzed in PBS or Tris-sucrose were stored at 2–8 °C and 20 °C for up to six months (180 days).

Overall, our data suggest that using a higher concentration of starting materials for LNP production improves long-term storage stability, both at refrigerated conditions and/or ambient temperatures, at least up to a lipid mixture concentration of 45 mg/mL. Results revealed that the effect of increased lipid mixture concentrations on improving long-term stability for LNPs stored at 2–8 °C was more pronounced in LNPs dialyzed in PBS compared to LNPs dialyzed in Tris-sucrose (Figure 11). At 2–8 °C storage, LNPs in PBS with lipid mixture concentrations of 45 and 70 mg/mL were more stable compared to LNPs of 15 mg/mL (Figure 11). However, for the same LNPs stored at 20 °C, stability only improved for LNPs with a lipid mixture concentration of 45 mg/mL but not for LNPs of 70 mg/mL. More specifically, LNPs in PBS stored at 2–8 °C with lipid mixture concentrations of 45 mg/mL and 70 mg/mL remained ≤120 nm and PDI ≤ 0.25 for at least 150 days (116 ± 41 nm, PDI 0.22 ± 0.08) and 180 days (95 ± 6 nm, PDI 0.21 ± 0.04), respectively (Supplementary Materials, Figure S2a,b). At 20 °C, LNPs in PBS of 45 mg/mL were stable for 60 days (99 ± 6 nm, PDI 0.20 ± 0.08), while LNPs in PBS of 70 mg/mL were stable for 1 day (72 ± 8, PDI 0.15 ± 0.05) (Supplementary Materials, Figure S2a,b). Meanwhile, LNPs in PBS with a lipid mixture concentration of 15 mg/mL stored at 2–8 °C were stable for 60 days (91 ± 8 nm, PDI 0.28 ± 0.02), whereas the same LNPs stored at 20 °C were stable for 21 days (88 ± 23 nm, PDI 0.23 ± 0.08) (Supplementary Materials, Figure S2a,b).

Meanwhile, for Tris-sucrose LNPs stored at 2–8 °C, stability improved with a lipid mixture concentration of 45 mg/mL, whereas LNPs of 15 mg/mL and 70 mg/mL displayed a similar stability profile (Figure 11). At 20 °C, stability improved for both LNPs of 45 mg/mL and 70 mg/mL by up to three weeks of storage (Figure 11). More specifically, at 2–8 °C, Tris-sucrose LNPs of 15 mg/mL were stable for 21 days (112 ± 4 nm, PDI 0.25 ± 0.02), LNPs of 45 mg/mL were stable for 90 days (119 ± 11 nm, PDI 0.26 ± 0.02), whereas LNPs of 70 mg/mL were stable for 28 days (111 ± 10 nm, PDI 0.22 ± 0.03) (Supplementary Materials, Figure S2c,d). However, at 20 °C, LNPs of 15 mg/mL were stable for 7 days (119 ± 11 nm, PDI 0.23 ± 0.01), whereas LNPs of 45 mg/mL and 70 mg/mL were stable for 28 days (118 ± 5 nm, PDI 0.21 ± 0.02 and 114 ± 5 nm, PDI 0.21 ± 0.02, respectively) (Supplementary Materials, Figure S2c,d).

## 4. Discussion

Our results showed that high lipid mixture concentrations slightly enhance EE and RNA recovery (Table 2), as well as zeta-potentials (Table 1 and Table 2), while simultaneously reducing bleb formation in SM-102 LNPs. The improved potency observed at higher buffer strength is primarily due to a more positively charged LNP surface that facilitates cellular uptake, combined with a high mRNA content within the LNPs that is required for efficient gene expression once internalized. Therefore, we suggest that bleb formation may not be the primary determinant of transfection efficiency; rather, RNA content and surface charge induced by buffer ionic strength play a more significant role [39]. Several other studies, including Brader et al. and Meulewaeter et al., have reported no clear correlation between bleb formation and increased transfection potency [47,48]. Additionally, we previously demonstrated that the formation of bleb-dissociated particles led to decreased expression both in vitro and in vivo [39]. In contrast to Liao’s study, where they achieved high-density LNPs (HDLNP) with elevated mRNA content via post-formulation centrifugation after dialysis [57], we demonstrated that high mRNA loading can be attained directly by increasing lipid mixture concentration during mixing, thereby avoiding potential changes either at the molecular level or in structural morphology that may result from high-shear centrifugation. While Liao et al. associated higher RNA content with more bleb formation on the LNPs and reduced transfection potency, their use of post-formulation fractionation may have introduced deviations in the N/P ratio for the HDLNPs [57], which could explain the reported decrease in transfection efficiency. Notably, they also reported that biodistribution was not affected by LNP morphology, indicating that bleb formation did not influence cellular uptake.

Moreover, our data in Figure 2 showed that LNPs prepared at high lipid mixture concentration formed fewer bleb structures yet displayed higher RNA recoveries (Table 2). Additionally, LNPs dialyzed in 300 mM Tris-sucrose exhibited a three-fold increase in transfection efficiency compared to LNPs in 10 mM PBS, aligning with Cheng et al., who reported that increased ionic strength is associated with enhanced transfection potency [58]. Although Cheng et al. attributed this effect to increased bleb formation, we believe this is a correlation rather than causation, with the effect dependent on the ionizable lipid used as well as buffer composition [39,58]. This is further supported by our observation that transfection efficiency also improved under low-strength buffer conditions (e.g., 10 mM PBS) when comparing LNPs prepared at different lipid mixture concentrations (Figure 9). Specifically, LNPs formulated at low lipid mixture concentration exhibited more bleb formation and lower transfection efficiency, whereas those prepared at high lipid mixture concentration showed fewer blebs and higher transfection efficiency (Figure 2 and Figure 9). These results suggest that the differences in transfection efficiency under low ionic strength conditions are primarily driven by lipid mixture concentration affecting the surface charge and mRNA content rather than bleb formation. While higher ionic strength buffers, such as 300 mM Tris-sucrose, enhance transfection efficiency, this improvement does not appear to be caused by increased bleb formation but is likely due to other factors such as changes in surface charge, as indicated by zeta-potentials.

Beyond the influence of the surface charge, we hypothesize that LNPs formulated at lipid mixture concentrations of 45 and 70 mg/mL may adopt a denser lipid matrix given the increased lipid and RNA loading under robust mixing conditions, while maintaining a consistent particle size. A more compact lipid arrangement—driven by a shrinkage of the LNP core after mRNA encapsulation [59]—could lead to a denser PEG chain alignment at the particle surface [60], potentially enhancing cellular uptake through two mechanisms: (i) preventing aggregation, thereby facilitating a more efficient uptake [4], or (ii) extending the circulation time in vivo, reducing immune cell recognition, as demonstrated by our findings in vivo (Figure 8). Note that the molar concentration of the PEG-lipid here was kept constant (1.5%) for all formulations, and the density of the arrangement of PEG chains discussed here is hypothetically assumed to be due to a denser packing of the lipid matrix rather than a higher PEG concentration, since higher concentrations of PEG decrease the in vivo efficiency [61]. However, this assumption should be further investigated using appropriate analytical tools, such as Atomic Force Microscopy (AFM) or Small-Angle X-ray Scattering (SAXS) to elucidate the structural organization of the surface layers and the packing density of the lipid matrix [59,60,62]. In a recent study, Kloczewiak et al. argued that, alongside LNP morphology, lipid packing influences the delivery efficiency of the mRNA into cells, thus affecting its expression levels [63].

In terms of storage stability, LNPs produced with a lipid mixture concentration of 70 mg/mL were highly influenced by both the storage temperature and buffer composition. For example, at 2–8 °C, stability improved substantially for LNPs in PBS of 70 mg/mL but was either lower at 20 °C and/or did not improve for LNPs in Tris-sucrose (Figure 11). Our results for LNPs in PBS of 15 mg/mL agreed with findings by Zhang et al., that SM-102-containing LNPs stored at 4 °C were stable for 8 weeks (Figure 11) [36]. However, we found that the same LNPs produced with higher lipid mixture concentrations of 45 and 70 mg/mL were stable for 24 weeks [36]. Further, although sucrose has been shown to improve the long-term stability of LNPs under frozen conditions, as well as improve gene expression (Figure 8 and Figure 9) [36], it did not improve the long-term stability of LNPs stored at 2–8 °C. On the contrary, LNPs in Tris-sucrose were less stable compared to LNPs in PBS. This may be due to the larger particle sizes of LNPs dialyzed in Tris-sucrose (Table 1 and Table 2). The higher ionic strength in the Tris-sucrose LNPs could have potentially reduced the repulsion between lipid head groups at the surface, affecting the lipid packing, thereby leading to the formation of larger particles [32,64]. In this line, the lipids in the larger Tris-sucrose LNPs are not as tightly packed, making them more susceptible to surface hydration and accelerating the aggregation process. In addition, the positive zeta-potentials of the Tris-sucrose LNPs, which are largely due to the protonated amine groups at the particle surface, hint towards thicker lipid membranes, which have been shown to correlate with increased membrane hydration [46]. In contrast, the PEG-lipids in the smaller PBS LNPs might be more compactly arranged at the surface, obstructing the hydration of the particle surface, and thus delaying the aggregation process.

However, it should be noted that the long-term stability of LNP-based formulations is heavily influenced by the structure of the encapsulated payload [65]. Various studies claim that the long-term instability of mRNA generally comes from its hydrolytic degradation as a result of water-containing pockets within the lipid matrix and/or other LNP morphologies [48,60,66]. Several strategies have been identified and implemented—including the commercially approved vaccines from Pfizer/BioNTech and Moderna—to optimize the stability of the mRNA structure [36,67,68].

## 5. Limitations of the Study

While this study offers valuable insights on how lipid mixture and payload concentrations affect LNP properties and in vivo transfection, it should be recognized that the specific lipid composition fundamentally dictates the pharmacokinetics; therefore, these outcomes may vary across different LNP models. Furthermore, while we reported the 6-month colloidal stability regarding particle size and PDI, an exhaustive assessment of other quality attributes—such as mRNA and lipid content, EE, mRNA potency, and morphological changes—was outside the current scope of this study. Further, to build upon these findings, incorporating high-resolution techniques like SAXS is recommended to provide deeper insights into internal matrix geometry and payload release kinetics. Finally, as the internal LNP environment significantly influences mRNA stability, investigating the relationship between various payload structures and long-term stability remains a vital direction for subsequent research.

## 6. Conclusions

LNPs have emerged as a powerful toolbox accelerating drug development at an unprecedented pace and effectively challenging the traditional decade-long timelines for bringing novel therapies to market. Our study demonstrated that LNPs can be produced at lipid mixture concentrations above 50 mg/mL when using a robust jet-impinging mixing technology, such as the FR-JET^®^ modular mixer. Results indicated that LNPs with consistent particle size could be produced across various lipid mixture concentrations and TFRs. The study confirmed that the preparation process preserved the integrity of encapsulated mRNA even when employing considerably high material concentrations and high TFRs. In vitro gene expression experiments indicated that LNPs with higher lipid mixture concentrations and higher mRNA recovery, as well as more positively charged surfaces, exhibited improved luciferase expression in HEK293 cells. Buffer composition also affected gene expression, with Tris-sucrose LNPs displaying a superior gene expression both in vitro and in vivo. In terms of LNP storage stability, our results demonstrated that LNP stability improved with increased lipid mixture concentrations up to 45 mg/mL, regardless of storage conditions. However, for LNPs at 70 mg/mL, it was observed that stability was more susceptible to both storage temperature and buffer composition. Notably, Tris-sucrose LNPs were less stable than LNPs in PBS, likely due to their larger size and increased surface hydration, leading to quicker aggregation. More importantly, LNPs formulated with high lipid mixture concentrations displayed improved gene expression in both in vitro and in vivo studies. In conclusion, our findings provide valuable insights into the preparation of LNPs at elevated starting material concentrations, demonstrating that process intensification can be achieved by adjusting this process parameter when prepared under efficient mixing without compromising product quality, while also boosting the biological performance of mRNA-LNP therapeutics.

## 7. Patents

The work reported in this manuscript has resulted in a patent application. Author B.S. is listed as an inventor on the related patent application, which is owned by the funder, Leon-Nanodrugs GmbH. The application details the optimized LNP formulation methods, specifically those using the proprietary FR-JET^®^ jet-impingement mixing technology (provided by Leon-Nanodrugs GmbH) to achieve enhanced in vivo gene expression and storage stability as described herein.

## Figures and Tables

**Table 1:** Poly(A)-LNPs formulated at a TFR of 15 mL/min over a range of lipid mixture concentrations. ZP denotes zeta-potential. Formulations dialyzed in PBS (*n* = 3). Formulations were dialyzed in Tris-sucrose (*n* = 1).

PBS					Tris-Sucrose			
Lipid Mix (mg/mL)	Size (nm)	PDI (−)	EE (%)	ZP (mV)	Size (nm)	PDI (−)	EE (%)	ZP (mV)
5	86 ± 6	0.08 ± 0.03	96 ± 1	−2 ± 1	/	/	/	/
15	82 ± 3	0.10 ± 0.02	96 ± 3	−2 ± 5	108	0.19	93	+25
30	84 ± 8	0.11 ± 0.04	96 ± 2	4 ± 0	112	0.20	98	+25
40	83 ± 8	0.11 ± 0.03	96 ± 2	8 ± 1	109	0.13	97	+27
50	80 ± 14	0.09 ± 0.05	96 ± 3	9 ± 4	102	0.15	98	+33
60	80 ± 8	0.08 ± 0.04	97 ± 2	10 ± 2	103	0.15	98	+28
70	76 ± 6	0.09 ± 0.02	98 ± 3	11 ± 3	103	0.15	97	+32

**Table 2:** Physico-chemical properties of poly(A)-LNPs prepared with lipid mixture concentrations of 15, 45, and 70 mg/mL. ZP denotes zeta-potential. Formulations were prepared in triplicates (*n* = 3).

PBS							Tris-Sucrose				
Lipid Mix (mg/mL)	TFR (mL/min)	Size (nm)	PDI (−)	EE (%)	Recovery (%)	ZP (mV)	Size (nm)	PDI (−)	EE (%)	Recovery (%)	ZP (mV)
15	30	66 ± 5	0.12 ± 0.03	93 ± 2	64 ± 10	−3 ± 4	94 ± 5	0.18 ± 0.03	92 ± 3	75 ± 15	+11 ± 3
	60	64 ± 3	0.11 ± 0.02	90 ± 1	62 ± 3	−2 ± 1	92 ± 6	0.19 ± 0.01	90 ± 0	78 ± 6	+17 ± 1
	80	69 ± 7	0.12 ± 0.01	91 ± 1	70 ± 5	−2 ± 0	98 ± 10	0.19 ± 0.04	92 ± 1	86 ± 8	+16 ± 3
45	30	63 ± 2	0.14 ± 0.02	93 ± 3	84 ± 20	+5 ± 5	100 ± 10	0.19 ± 0.01	94 ± 1	96 ± 8	+14 ± 9
	60	66 ± 12	0.13 ± 0.01	94 ± 3	85 ± 8	+6 ± 4	95 ± 17	0.21 ± 0.01	95 ± 3	96 ± 7	+17 ± 7
	80	62 ± 8	0.15 ± 0.03	93 ± 2	82 ± 9	+6 ± 3	104 ± 8	0.19 ± 0.01	93 ± 7	88 ± 16	+18 ± 6
70	30	71 ± 1	0.17 ± 0.03	92 ± 3	88 ± 6	+9 ± 9	96 ± 4	0.18 ± 0.03	94 ± 1	99 ± 1	+17 ± 6
	60	74 ± 3	0.12 ± 0.01	93 ± 1	88 ± 10	+6 ± 3	91 ± 7	0.19 ± 0.02	95 ± 3	90 ± 17	+29 ± 6
	80	63± 6	0.14 ± 0.03	95 ± 3	90 ± 17	+3 ± 3	93 ± 10	0.19 ± 0.05	94 ± 3	90 ± 0	+27 ± 9

## Data Availability

The datasets generated and analyzed during the current study are not publicly available as they contain proprietary information belonging to LEON-nanodrugs GmbH. However, the data are available from the corresponding author upon reasonable request, subject to a formal non-disclosure agreement.

## Supplementary Materials

The following supporting information can be downloaded at: https://www.mdpi.com/article/10.3390/pharmaceutics18010050/s1, Figure S1: Quantification of DiR indicating in vivo biodistribution (left), and luciferase expression in the liver (right). mRNA-LNPs were prepared with a lipid mixture concentration of 15 mg/mL, 45 mg/mL and 70 mg/mL and dialyzed against Tris-sucrose or PBS. Quantification of bioluminescence in the liver; Figure S2: Particle size (A) and PDI (B) of poly(A)-LNPs dialyzed in PBS; Particle size (C) and PDI (D) of poly(A)-LNPs dialyzed in Tris-sucrose. LNPs were stored at 2–8 °C and 20 °C and measured on DLS at predetermined timepoints over 6 months. Dotted lines indicate stability threshold of 120 nm and PDI ≤ 0.2, whereas X indicates that samples were unstable and not measured. Error bars represent the standard deviation between formulation replicates (*n* = 3).

## Abbreviations

The following abbreviations are used in this manuscript:

AAV	Adeno-associated virus
ALC-0315	[(4-Hydroxybutyl)azanediyl]di(hexane-6,1-diyl) bis(2-hexyldecanoate)
CGT	Cell and gene therapies
CKK-E12	3,6-Bis [4-[bis (2-hydroxydodecyl)amino]butyl]-2,5-piperazinedione
cryoTEM	Cryogenic transmission electron microscope
DEPC	Diethyl pyrocarbonate
DiR	1,1′-Dicotadecyl-3,3,3′,3′-tetramethylindocarbocyanine iodide
DLS	Dynamic light scattering
DMG-PEG2000	1,2-dimyristoyl-rac-glycero-3-methoxypolyethylene glycol-2000
DSPC	1,2-Distearoyl-sn-glycero-3-phosphocholine
EE	Encapsulation efficiency
Fluc-mRNA	Firefly luciferase mRNA
FRR	Flow rate ratio
IVIS	In vivo imaging system
i.m.	Intramuscular
kDa	Kilodalton
LN_2_	Liquid nitrogen
LNP	Lipid nanoparticles
MEM	Minimal essential medium
ML	Machine learning
mRNA	Messenger RNA
MWCO	Molecular weight cut-off
N/P	Nitrogen/Phosphate
NLC	Nanostructured lipid carriers
PBS	Phosphate-buffered saline
PDI	Polydispersity index
Poly(A)	Polyadenosine
RLU	Relative light unit
SM-102	Heptadecan-9-yl 8-((2-hydroxyethyl)[6-oxo-6-(undecyloxy)hexyl]amino) octanoate
SLN	Solid lipid nanoparticles
TFF	Tangential flow filtration
TFR	Total flow rate
ZP	Zeta-potential
