# Supplementary material for: Boosting LNP Performance: Higher Concentrations of Lipid Mixtures Improve In Vivo Gene Expression and Storage Stability
Source: Pharmaceutics. 2025 Dec 30;18(1):50. doi: 10.3390/pharmaceutics18010050 (PMC12845254; doi:10.3390/pharmaceutics18010050)
Supplement: Supplementary file 1 [file pharmaceutics-18-00050-s001.zip › pharmaceutics-4046030-supplementary.pdf]

Supplementary Material

Boosting LNP Performance: Higher Concentrations of Lipid Mixture Improve *In Vivo* Gene Expression and Storage Stability

Blerina Shkodra[1†*], Ashish Muglikar[2†], Janani Thangapandian[1], Matthias Schumacher[1], Burcu Binici[2], Yvonne Perrie[2]

1) Leon-nanodrugs GmbH, Am Klopferspitz 19, 82152 Planegg, Germany; j.thangapandian@leon-nanodrugs.com; m.schumacher@leon-nanodrugs.com
2) Strathclyde Institute of Pharmacy and Biomedical Sciences, University of Strathclyde, 161 Cathedral Street, Glasgow G4 0RE, UK; muglikar11ashish@gmail.com; burcu.eryilmaz@strath.ac.uk; yvonne.perrie@strath.ac.uk;
** Correspondence: b.shkodra@leon-nanodrugs.com; Tel.: +49-15221096740*
† These authors contributed equally to this work

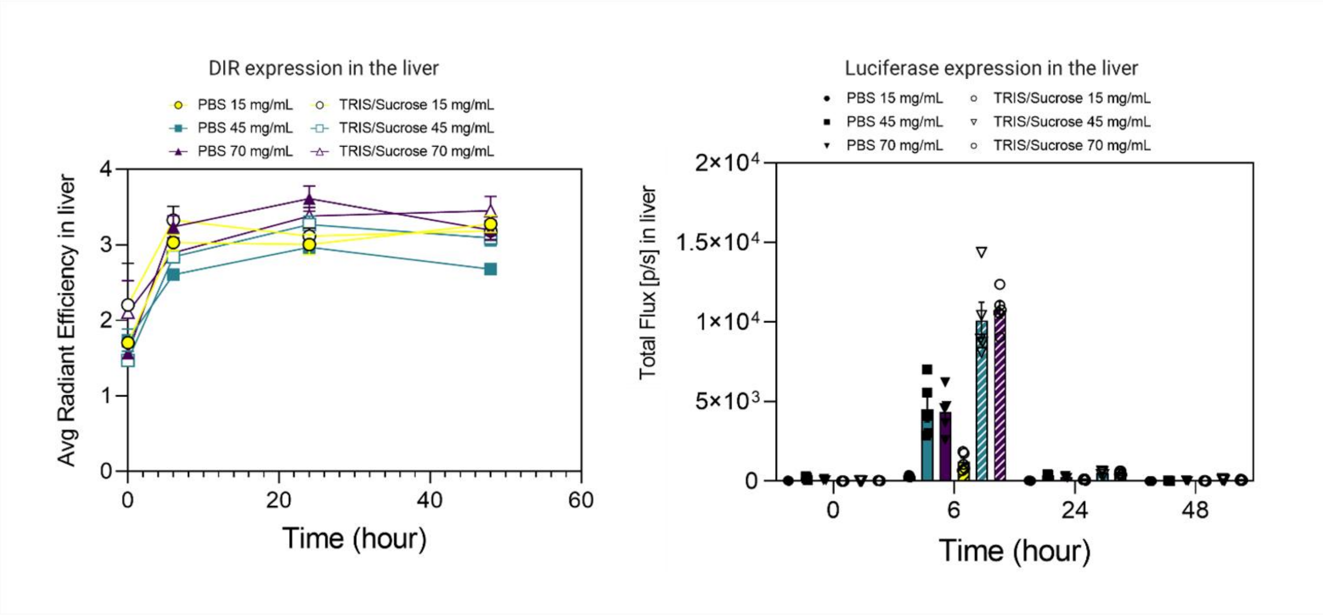

Figure S1. *Quantification of DiR indicating in vivo biodistribution (left), and luciferase expression in the liver (right). mRNA-LNPs were prepared with a lipid mixture concentration of 15 mg/mL, 45 mg/mL and 70 mg/mL and dialyzed against Tris-sucrose or PBS. Quantification of bioluminescence in the liver.*

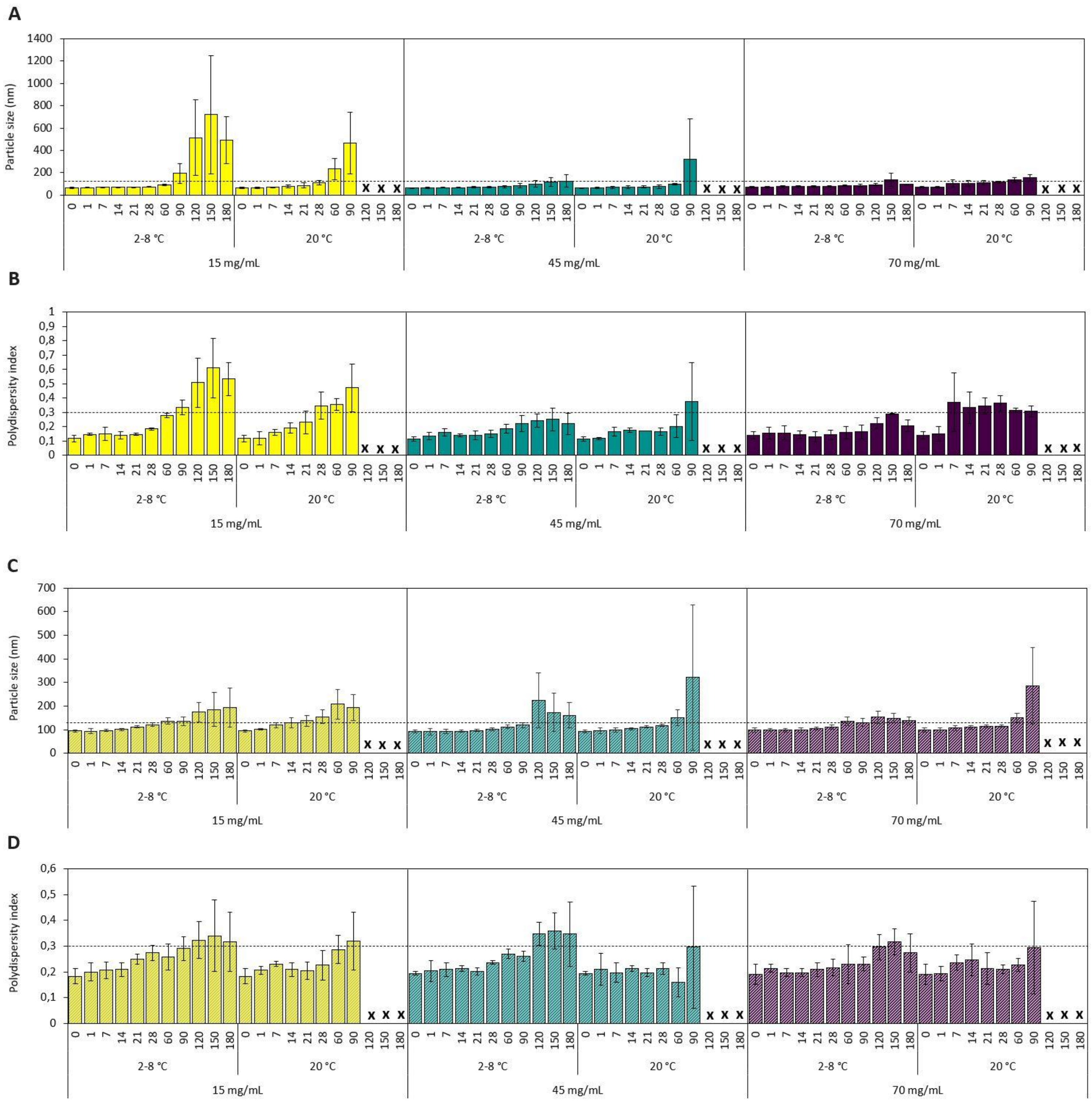

Figure S2. *Particle size (**A**) and PDI (**B**) of poly(A)-LNPs dialyzed in PBS; Particle size (**C**) and PDI (**D**) of poly(A)-LNPs dialyzed in Tris-sucrose. LNPs were stored at 2–8 °C and 20 °C and measured on DLS at predetermined timepoints over 6 months. Dotted lines indicate stability threshold of 120 nm and PDI ≤0.2, whereas **X** indicates that samples were unstable and not measured. Error bars represent the standard deviation between formulation replicates (n=3).*
